# Clinical examination for identifying low-risk pregnancies suitable for expectant management beyond 40–41 gestational weeks: maternal and fetal outcomes

**DOI:** 10.1007/s00404-024-07869-5

**Published:** 2024-12-17

**Authors:** Gulim Murzakanova, Sari Räisänen, Anne Flem Jacobsen, Branka M. Yli, Tiril Tingleff, Katariina Laine

**Affiliations:** 1 Department of Obstetrics, Oslo University Hospital, University of Oslo, Pb 4965, Nydalen, 0424 Oslo, Norway; 2 Institute of Clinical Medicine, Faculty of Medicine, University of Oslo, Oslo, Norway; 3 Laurea University of Applied Sciences, Vantaa, Finland; 4 Department of Obstetrics, Haukeland University Hospital, Bergen, Norway; 5 Norwegian Research Centre for Women’s Health, Oslo University Hospital, Oslo, Norway

**Keywords:** Labor induction, Term births, Natural childbirth, Gestational age, Post-term pregnancy, Late-term pregnancy

## Abstract

**Purpose:**

There is an ongoing discussion on whether the benefits of term elective labor induction outweigh its potential risks. This study evaluated the utility of a comprehensive clinical examination in identifying low-risk pregnancies suitable for expectant management beyond gestational age 40‒41 weeks and compared their outcomes with earlier labor induction by indication.

**Methods:**

Pregnant women (*n* = 722) with ≥ 40 + 0 gestational weeks referred to a tertiary hospital were included in this prospective cohort. The study population was divided into the primary induction group (induction before 42 + 0 gestational weeks) and the expectant management group (spontaneous labor onset or induction at 42 + 0 gestational weeks), by decision based on a primary consultation. The Chi-square test and logistic regression were applied. The outcome measures were composite adverse fetal outcome (admission to a neonatal intensive care unit, metabolic acidosis, or Apgar score < 7 at 5 min), treatment with intrapartum antibiotics, intrapartum maternal fever ≥ 38 °C, intrapartum cesarean section, and postpartum hemorrhage ≥ 1500 ml.

**Results:**

The main outcome measures did not differ significantly between the primary induction group (*n* = 258) and the expectant management group (*n* = 464): composite adverse fetal outcome (OR = 2.29, 95% CI = 0.92–5.68; *p* = 0.07), intrapartum cesarean section (OR = 1.00, 95% CI = 0.64–1.56; *p* = 1.00), postpartum hemorrhage ≥ 1500 ml (OR = 1.89, 95% CI = 0.92–3.90; *p* = 0.09), intrapartum maternal fever ≥ 38 °C (OR = 1.26, 95% CI = 0.83–1.93; *p* = 0.28), or treatment with intrapartum antibiotics (OR = 1.25, 95% CI = 0.77–2.02; *p* = 0.37).

**Conclusion:**

A comprehensive clinical examination at 40‒41 gestational weeks can identify pregnancies that might be managed expectantly until 42 gestational weeks obtaining similar outcomes to those induced earlier.

## What does this study add to the clinical work?

**Table:** 

A thorough clinical examination at term can effectively differentiate between pregnancies that require labor induction and those that are suitable for expectant management until 42 gestational weeks.	

## Introduction

Previous studies have shown that the risk of adverse fetal outcomes [1, 2] including stillbirth [3] is higher in post-term pregnancies than term pregnancies. With the aim of preventing stillbirth, the prevalence of non-medically indicated labor induction has been increasing worldwide [4, 5].

The management of pregnancies at gestational age (GA) ≥ 40 weeks varies both between and within countries, with labor induction mostly offered at GA 41‒42 weeks [6–8]. Norway offers labor induction at GA 42 + 0 weeks in uncomplicated pregnancies [9].

The ARRIVE randomized controlled trial (RCT) in 2018 compared early induction at GA 39 + 0 to 39 + 4 weeks with expectant management followed by induction no later than GA 42 + 2 weeks among low-risk nulliparous women [10]. That study found no significant difference in adverse perinatal outcomes between the groups. However, it did observe that the rate of cesarean sections (CSs) was lower in the early induction group than the expectant management group. Another RCT, the SWEPIS study from 2019 [11], compared labor induction at GA 41 weeks versus GA 42 weeks in low-risk pregnancies. The study was stopped prematurely because a significantly higher perinatal mortality was observed in the expectant management group. These studies have led to many international guidelines changing the recommendations to the more-liberal approach, offering labor induction at earlier GA than 42 weeks [6, 12].

Two previous Swedish studies demonstrated that including a routine ultrasound examination at GA 41 weeks reduced the proportion of adverse fetal outcomes in post-term births to make the proportion comparable to that in term births [13]. Moreover, a routine ultrasound examination improved identification of fetuses at risk [14].

Some women report negative or even traumatic experiences with the induction of labor, leading them to prefer avoiding this procedure in low-risk pregnancies where there is no medical indication [15]. It is also important to note that fetal organs, including the brain, continue to grow and develop beyond GA 40 weeks [16].

The aim of the present study was to evaluate the utility of a comprehensive clinical examination in identifying low-risk pregnancies suitable for expectant management beyond GA 40‒41 weeks and to compare their outcomes with earlier labor induction by indication.

## Participants, ethics, and methods

### Design and ethics

This prospective cohort study was a quality assurance project conducted at Oslo University Hospital (OUH), a tertiary maternity hospital in Norway and was approved by the Institutional Personal Data Officer at the same hospital.

### Setting and population

All antenatal care is provided free of charge in Norway. For uncomplicated pregnancies, this care is managed by a midwife or a general practitioner in the primary health care system. The individual is referred to a maternity hospital if a consultation with a specialist is deemed necessary. During the study period, the Norwegian national guidelines recommended an initial consultation at the outpatient clinic of the maternity hospital for all women who reached GA 41 + 2 weeks [9]. Women with risk factors such as comorbidity, advanced age, or obesity, were offered a primary consultation at GA 40 + 0 weeks.

**Fig. 1:**
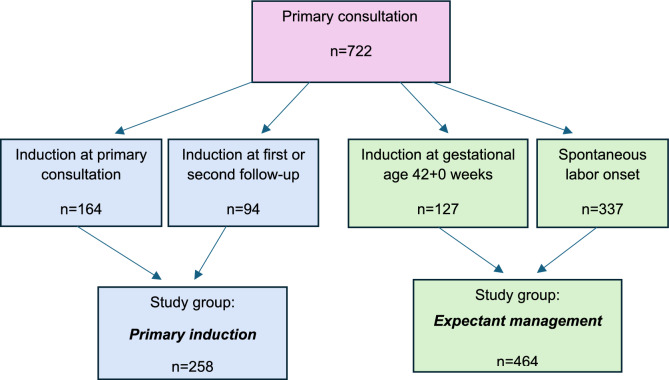
Flow chart of study enrollment

This study included all pregnant women referred to the maternity hospital for a primary consultation at GA ≥ 40 + 0 weeks between January 28 and September 4, 2021. No exclusion criteria were applied. The study population was divided into two groups: (1) primary induction group and (2) expectant management group. The primary induction group included individuals who were induced before GA 42 + 0 weeks due to indications identified during the primary consultation or during the serial follow-ups. The expectant management group comprised individuals who experienced spontaneous labor onset as well as those who were induced at GA 42 + 0 weeks (Fig. 1).

### Description of the intervention

The primary consultation included a thorough clinical examination with cardiotocography (CTG), blood pressure (BP) measurement, urinary dipstick testing, an assessment of the cervical ripening status, and an ultrasound examination for determining the estimated fetal weight (EFW) and amniotic fluid volume. The women were also asked about their perception of normal fetal movements. The umbilical artery Doppler (UAD) pulsatility index (PI) was measured in most cases, but this was optional.

Labor induction was recommended if abnormalities were found, such as oligohydramnios, EFW < 5th percentile, abdominal circumference (AC) ≤ 5th percentile, or a discrepancy ≥ 14 days between the due date based on the last menstrual period (LMP) and the due date based on the second-trimester ultrasound examination. If no abnormalities or risk factors were identified, expectant management was offered with serial antenatal follow-ups every second or third day until labor induction at GA 42 + 0 weeks. The follow-up consultations consisted of the clinical examination as described above except for the determining of the EFW.

### Data collection and extraction based on the primary consultation

Information on the clinical decision to perform either induction (along with the indication) or expectant management was collected prospectively. Data on labor onset, events during labor and birth, and maternal and neonatal outcomes were gathered from individual patient records. The accuracy of the findings from the clinical examinations conducted in the primary consultation and in the follow-ups was verified after the birth.

Abnormal CTG findings, abnormal UAD PI, or reduced fetal movements were grouped into one variable because they represent fetal compromise affecting the decision about labor induction. The assessment of the fetal movement pattern was based on the information given by the mother and observations of fetal movements during the ultrasound examination. CTG were interpreted according to modified FIGO guidelines [17], and abnormalities in the UAD PI were defined as PI > 95th percentile according to Acharya et al. [18].

EFW < 5th percentile, AC ≤ 5th percentile, small for gestational age (SGA) (EFW < 5th percentile for GA), and suspicion of intrauterine growth restriction (IUGR) (EFW deviating from or a reduction in an expected fetal growth pattern) were defined in accordance with the reference range by Johnsen et al. [19]. Antepartum oligohydramnios was defined as a single deepest pocket of amniotic fluid ≤ 2 cm or amniotic fluid index ≤ 5 cm measured during an ultrasound examination.

The diagnosis of gestational hypertension (BP ≥ 140/90 mmHg at GA > 20 weeks), and preeclampsia (BP ≥ 140/90 mmHg and proteinuria at GA > 20 weeks) were based on the findings of the primary consultation which did not initially include laboratory test results. Preeclampsia and gestational hypertension were merged into one variable and used for descriptive purpose to list labor indications determined during the primary consultation.

Prelabor rupture of membranes included all cases of spontaneous membrane rupture at least 24 h before birth. Polyhydramnios was defined as a single deepest pocket of amniotic fluid ≥ 8 cm or amniotic fluid index ≥ 25 cm.

### Birth outcomes

An adverse fetal outcome was a composite variable including admission to a neonatal intensive care unit (NICU), metabolic acidosis, or Apgar score < 7 at 5 min. The main maternal adverse outcomes were treatment with intrapartum antibiotics, intrapartum maternal fever ≥ 38 °C, intrapartum CS, and postpartum hemorrhage ≥ 1500 ml.

Intrapartum oligohydramnios (subjective assessment of a reduced amount of amniotic fluid after membrane rupture) and NICU admission (admission of the neonate to NICU for observation or treatment for any reason) were documented during labor or after birth by the attending midwife. Metabolic acidosis was defined as umbilical artery pH < 7.00 and base deficit in extracellular fluid ≥ 12 mmol/L [9], as determined in an acid–base analysis of the umbilical cord blood. All cases with metabolic acidosis were validated according to the criteria by Kro et al. [20]. Apgar scores were determined and documented by the attending midwife, except in complicated births when this was done by a neonatologist.

Intrapartum maternal fever was defined as body temperature ≥ 38 °C measured in the rectum during labor, treatment with intrapartum antibiotics as treatment of the mother during labor with one or more doses of systemic antibiotics, and postpartum hemorrhage ≥ 1500 ml as hemorrhage occurring after birth or until hospital discharge.

### Maternal and fetal demographics and characteristics

The specific data on maternal and fetal demographics as well as pregnancy and birth characteristics included in the analyses were chosen based on the previous studies [1, 2].

Maternal age was categorized into five groups: ≤ 24, 25–29, 30–37, 38–39, and ≥ 40 years. The last two age categories were chosen due to labor induction being recommended for women aged > 38 years in the Norwegian national guidelines and for those aged ≥ 40 years according to the local guideline at OUH. Body mass index (BMI) was calculated based on maternal height and weight at the first antenatal visit either as the prepregnancy or first-visit BMI, and categorized into five groups: underweight (< 18.5 kg/m^2^), normal weight (18.5–24.9 kg/m^2^), overweight (25–29.9 kg/m^2^), moderately obese (30–34.9 kg/m^2^), and severely obese (> 35 kg/m^2^). Based on the number of previous births, parturients were classified as nulliparous (no previous births) or parous (number of previous births ≥ 1).

### Statistical analyses

Demographics, maternal, fetal, and obstetric outcomes, and indications for labor induction are presented as numbers and percentages. Continuous data were categorized. The Chi-square test with a significance level of 5% was used to test differences between the study groups. The privacy rights of the involved individuals according to the General Data Protection Regulation of the European Union were protected using < 5 to indicate groups with fewer than five cases in the descriptive statistics.

Logistic regression analyses were performed to determine crude odds ratios (ORs) for associations between the study groups (with the primary induction group as the reference group) and fetal and maternal outcomes. ORs are reported with 95% confidence intervals (CIs).

In 3.5% of cases (25 of 722), it was not possible to determine the discrepancy between the LMP-based due date and the ultrasound-based due date due to missing information on due date. In 7.8% of cases (56 of 722), the acid–base analysis was not available. The missing-information rate was < 1% for all other variables.

All statistical analyses were performed using SPSS software (version 29, IBM Corporation, Armonk, NY). The included women were not involved in the planning, design, or conduct of this study.

## Results

The study population comprised 722 individuals: 258 in the primary induction group and 464 in the expectant management group (Fig. 1). Table 1 reports the demographic and birth characteristics of the individuals in the two study groups. The prevalence rates of maternal age ≥ 40 years, BMI > 35 kg/m^2^, previous CS, EFW < 5th percentile, AC ≤ 5th percentile, antepartum and intrapartum oligohydramnios, and birthweight < 5th percentile were significantly higher in the primary induction group than the expectant management group (Table 1).

**Table 1:** Characteristics of the study cohort in the primary induction group (induction at GA < 42 weeks) and the expectant management group (spontaneous labor onset or induction at GA ≥ 42 weeks)

Variable	Primary induction group	Expectant management group
	*n* = 258	n = 464
	*n* (%)	*n* (%)
GA at primary consultation		
< 41 + 2 weeks	166 (64.34)	183 (39.44)
≥ 41 + 2 weeks	92 (35.66)	281 (60.56)
GA at birth		
40 + 0 to 40 + 6 weeks	38 (14.73)	45 (9.70)
41 + 0 to 41 + 6 weeks	178 (68.99)	243 (52.37)
≥ 42 + 0 weeks	42 (16.28)	176 (37.93)
Maternal age, years		
≤ 24	8 (3.10)	5 (1.08)
25–29	39 (15.12)	95 (20.47)
30–37	141 (54.65)	303 (65.30)
38–39	28 (10.85)	37 (7.97)
≥ 40	42 (16.28)	24 (5.17)
BMI, kg/m^2^		
< 18.5	5 (1.95)	14 (3.03)
18.5–24.9	162 (63.04)	334 (72.29)
25–29.9	62 (24.12)	82 (17.75)
30–34.9	21 (8.17)	31 (6.71)
> 35	7 (2.72)	< 5^a^
Parity		
Nulliparous	150 (58.14)	271 (58.41)
Parous	108 (41.86)	193 (41.59)
Previous CS	22 (8.53)	13 (2.80)
Discrepancy ≥ 14 days between LMP dating and ultrasound dating	9 (3.49)	5 (1.08)
Missing LMP dating or ultrasound dating	6 (2.33)	19 (4.09)
EFW < 5th percentile	6 (2.33)	< 5^a^
AC ≤ 5th percentile	9 (1.94)	16 (6.20)
Number of follow-ups		
1	74 (28.68)	121 (26.08)
2	20 (7.75)	17 (3.66)
Antepartum oligohydramnios	33 (12.79)	11 (2.37)
Intrapartum oligohydramnios	32 (12.40)	27 (5.82)
Intrapartum maternal fever ≥ 38 °C	37 (14.34)	81 (17.46)
Intrapartum antibiotics	27 (10.46)	59 (12.72)
Birth mode		
Vaginal birth	216 (83.73)	400 (86.18)
Intrapartum CS	35 (13.57)	63 (13.58)
Prelabor CS	7 (2.71)	< 5^a^
Birthweight < 5th percentile	19 (7.36)	11 (2.37)
Composite adverse fetal outcome	6 (2.33)	24 (5.17)
Apgar score < 7 at 5 min	< 5^a^	6 (1.29)
Metabolic acidosis	0	< 5^a^
NICU admission	5 (1.94)	20 (4.31)
Postpartum hemorrhage ≥ 1500 ml	10 (3.88)	33 (7.11)

*AC* abdominal circumference, *BMI* body mass index, *CS* cesarean section, *EFW* estimated fetal weight, *GA* gestational age, *LMP* last menstrual period, *NICU* neonatal intensive care unit

^a^Exact number of cases not listed to protect privacy rights according to the General Data Protection Regulation of the European Union

**Fig. 2:**
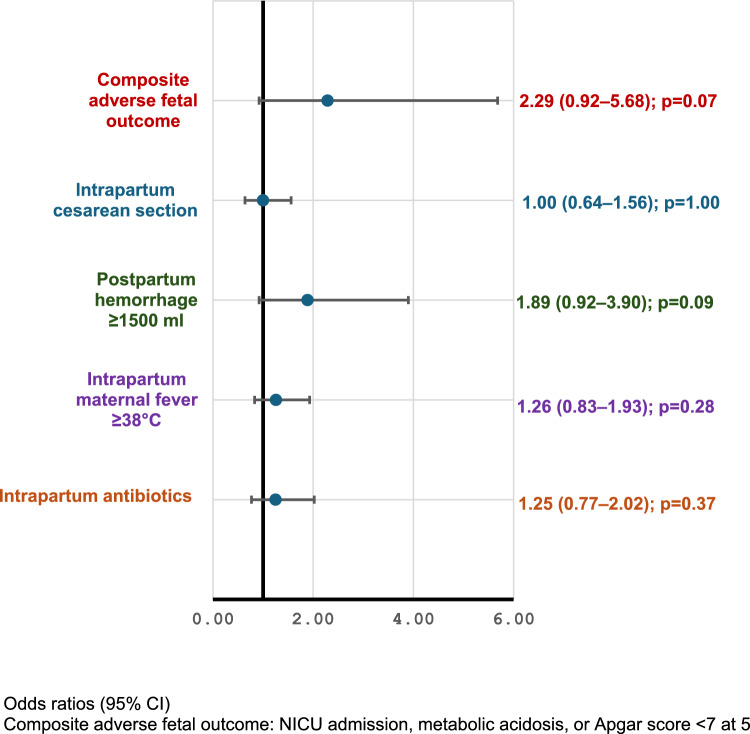
Crude odds ratios for main fetal and maternal outcomes for expectance group (spontaneous labor onset or induction at gestational age ≥ 42 weeks) compared to primary induction group (induction at gestational age < 42 weeks)

A crude logistic regression analysis revealed no significant intergroup differences in the composite adverse fetal outcome (OR = 2.29, 95% CI = 0.92–5.68; *p* = 0.07) or in maternal outcomes such as intrapartum CS (OR = 1.00, 95% CI = 0.64–1.56; *p* = 1.0), postpartum hemorrhage ≥ 1500 ml (OR = 1.89, 95% CI = 0.92–3.90; *p* = 0.09), intrapartum maternal fever ≥ 38 °C (OR = 1.26, 95% CI = 0.83–1.93; *p* = 0.28), or intrapartum antibiotics (OR = 1.25, 95% CI = 0.77–2.02; *p* = 0.37) (Fig. 2).

Since there were no significant differences between the groups in the crude analysis, a multivariable logistic regression analysis was not indicated.

The most frequent indication for labor induction was post-term pregnancy (36.6%), followed by maternal age ≥ 40 years (12.0%), oligohydramnios (11.4%), SGA or suspected IUGR (10.9%), abnormal CTG findings or abnormal UAD PI or reduced fetal movements (6.4%), maternal anxiety or a preference for induction (6.2%), and hypertension or preeclampsia (5.2%) (Table 2).

**Table 2:** Indications for labor induction (385 of 722 subjects)

Variable	N (%)
Indications recommended by guidelines for post-term pregnancies	
Post-term pregnancy	141 (36.6)
SGA or suspected IUGR	42 (10.9)
Oligohydramnios	44 (11.4)
Maternal age ≥ 40 years	46 (12.0)
Discrepancy ≥ 14 days between LMP dating and ultrasound dating	< 5^a^
Abnormal CTG findings, abnormal UAD PI, or reduced fetal movements	25 (6.4)
Maternal anxiety or preference for induction	24 (6.2)
Hypertension or preeclampsia	20 (5.2)
Polyhydramnios	5 (1.3)
PROM ≥ 24 h	12 (3.1)
Other^b^	24 (6.2)

*CTG* cardiotocography, *IUGR* intrauterine growth restriction, *PI* pulsatility index, *PROM* prelabor rupture of membranes, *SGA* small for gestational age, *UAD* umbilical artery Doppler

^a^Exact number of cases not shown to protect privacy rights according to the General Data Protection Regulation of the European Union

^b^High BMI, large for gestational age or large fetus, breech presentation, gestational diabetes or diabetes type 2, transverse lie, meconium-stained amniotic fluid, complicated obstetric history, vaginal bleeding, and reduced amniotic fluid

The antenatal detection rate of a low birthweight at < 5th percentile was 23.3% when determined based on the EFW, and 43.3% when based on an antenatal AC of < 5th percentile. The detection rate of oligohydramnios was 39.0% (Table 3).

**Table 3:** Antenatal detection rates (%) of birthweight < 5th percentile and oligohydramnios by ultrasound in the study population

Variable	Birthweight < 5th percentile (*n* = 30)	Detection rate
Estimated fetal weight < 5th percentile (*n* = 7)	7/30	23.3%
Abdominal circumference ≤ 5th percentile (*n* = 25)	13/30	43.3%
	Intrapartum oligohydramnios (n = 59)	
Antepartum oligohydramnios (*n* = 44)	23/59	39.0%

## Discussion

### Main findings

This prospective observational study utilizing real-world data found no significant differences in adverse maternal or fetal outcomes between the study groups. These findings indicate that, when a thorough clinical examination uncovers no abnormalities, it appears safe to proceed with expectant management with serial antenatal follow-up until performing labor induction at GA 42 weeks. This result is in accordance with the low perinatal mortality rate in Norway, where there is universal access to maternity care services for all pregnant individuals [21].

### Strengths and limitations

The primary strength of this study lies in its prospective design, which allowed for data collection in an unselected, real-world population. All the included individuals underwent standardized and meticulous clinical examinations throughout the study period, with the findings being carefully recorded. These comprehensive clinical examinations facilitated the ability to select pregnancies at risk of early induction, while uncomplicated pregnancies could continue to GA 42 + 0 weeks. In addition, extensive data on maternal demographics as well as on pregnancy and birth characteristics and outcomes were gathered from patient records that were prospectively collected during the pregnancies.

The study also had some limitations, including insufficient statistical power to assess rare outcomes such as perinatal mortality, and the inability to determine causality due to its observational design. The results suggest that there may have been more adverse outcomes in the expectant management group; however, the differences were not statistically significant between the study groups.

### Interpretation

Our findings align with those of a previous Swedish study analyzing real-world data that demonstrated that incorporating routine ultrasound in the follow-up examinations at GA 41 weeks reduced the risk of adverse fetal outcomes in post-term pregnancies, making them comparable to term births [13]. Another study from Sweden demonstrated that routine ultrasound examination aids in identifying SGA fetuses, thereby improving their outcomes [14]. In our study, birthweight < 5th percentile was detected antenatally in 23.3% of cases when determined based on the EFW, and in 43.3% when based on an AC of < 5th percentile, which is in line with previous studies [13, 22]. Antenatal SGA detection can be increased by enhancing skills in obstetric ultrasound training through online learning platforms and e-learning methods that include innovative simulation technologies [23]. We could not compare our result of oligohydramnios being present in 39.0% of cases with other results since we did not find any other studies that have compared antepartum and intrapartum oligohydramnios.

Our findings are also consistent with a Norwegian RCT from 2007 [24] that found no difference in neonatal morbidity or mode of birth between the group induced at GA 41 weeks and the group managed with expectant management and labor induction at GA 42 weeks.

Our study differed significantly from the ARRIVE [10] and SWEPIS [11] RCTs as they focused solely on low-risk women and pregnancies in experimental settings, while our study encompassed a “real-world” population without applying any exclusion criteria. In our study, the management plans were based on findings from comprehensive clinical examinations, thereby distinguishing between low- and high-risk pregnancies. In contrast, such complete and comprehensive clinical examinations were not routinely performed in the SWEPIS study, and the mortality occurred among the study participants who were not followed up with CTG or ultrasound examination after the study enrollment. Also, since the study was discontinued, it is discussible whether it had enough statistical power to assess mortality. Furthermore, the population in the ARRIVE study was notably younger, with a median maternal age of just 23–24 years, which may reduce its generalizability to the “real-world” population.

The optimal time point for labor induction beyond term has been challenging to determine, and both maternal, neonatal, and children’s long-term outcomes are worth to be considered when such decisions are being made. A study from the Netherlands [16] found a linear association between total brain volume and morphometry at 10 years of age and the GA at birth. This association remained linear when the analysis was restricted to the term-born children. The brain is still growing and developing after GA 40 weeks, and there is an association between brain volume and intelligence [25], thus unnecessary induction without medical indications should be avoided. Increased risks of long-term complications have been reported for all major neuropsychiatric disorders [26], pediatric respiratory hospitalizations [27], and low neurocognitive scores of children [28] for births at GA 37‒38 weeks compared with later births. These long-term implications for the child’s health, therefore, needed to be considered when deciding about labor induction for nonmedical indications and counseling mothers at an earlier GA. Neither the ARRIVE nor the SWEPIS study, mentioned above, assessed or discussed the potential long-term consequences of birth at a given GA for the child.

We agree with Azria et al. and Seijmonsbergen-Schermers et al. [29, 30] regarding ethical issues of non-medically indicated labor induction. Medical staff recommending labor induction puts the pregnant woman in a difficult situation by potentially reducing her empowerment and inhibiting her right to have a physiologic labor and birth. Assessments of the experiences with labor induction have revealed that many women feel that they were not properly involved in decision-making, had a reduced sense of ownership, felt that their social needs were not met due to them feeling forgotten or alone in the hospital, and experienced little privacy during the labor induction process [31]. Many have also experienced powerlessness, a lack of control, and periods of anxiety, pain, and discomfort during the labor induction process [15]. These ethical and empowerment issues and the need to determine whether findings of the ARRIVE study are generalizable to other countries prompted the French ARRIVE study to be performed (NCT04799912) [32]. This has triggered a debate about whether the world is about to “medicalize the childbirth” and impair the right of women to experience the normal physiologic process of childbirth [33, 34].

Labor inductions require resource commitments, and unnecessary inductions can be a burden on maternity units and reduce the availability of hospital beds to other groups in need. Recent studies found that outpatient clinics were used more by those in the expectant management group than the induction group [35, 36], while inpatient intrapartum care costs were higher in the latter group [36]. Furthermore, Hersh et al. [37] showed that the hospitalization costs for both women and neonates were higher for the elective induction of labor than for spontaneous labor.

The probability of spontaneous labor onset increases continuously at term weeks [38]. Therefore, an appropriate expectant management policy can increase the benefits of women experiencing normal labor and saving health care resources for groups that need it most by avoiding unnecessary labor inductions.

## Conclusion

Maternal and fetal outcomes were comparable between the study groups. These results indicate that a thorough clinical examination at term can effectively distinguish between pregnancies requiring labor induction before GA 42 weeks and those suitable for expectant management until labor induction at GA 42 weeks.

## Data Availability

Due to the sensitive nature of the data and according to Norwegian research legislation and regulations, data sharing and availability is not possible.
